# Hemifacial Spasm Caused by Vascular Compression in the Cisternal Portion of the Facial Nerve: Report of Two Cases with Review of the Literature

**DOI:** 10.1155/2019/8526157

**Published:** 2019-01-01

**Authors:** Byung-chul Son, Hak-cheol Ko, Jin-gyu Choi

**Affiliations:** ^1^Department of Neurosurgery, Seoul St. Mary's Hospital, College of Medicine, The Catholic University of Korea, Seoul, Republic of Korea; ^2^Catholic Neuroscience Institute, College of Medicine, The Catholic University of Korea, Seoul, Republic of Korea; ^3^Department of Neurosurgery, Kyung Hee University Hospital at Gangdong, Kyung Hee University, Seoul, Republic of Korea; ^4^Department of Neurosurgery, Yeouido St. Mary's Hospital, College of Medicine, The Catholic University of Korea, Seoul, Republic of Korea

## Abstract

Although primary hemifacial spasm (HFS) is mostly related to a vascular compression of the facial nerve at its root exit zone (REZ), its occurrence in association with distal, cisternal portion has been repeatedly reported during the last two decades. We report two patients with typical HFS caused by distal neurovascular compression, in which the spasm was successfully treated with microvascular decompression (MVD). Vascular compression of distal, cisternal portion of the facial nerve was identified preoperatively in the magnetic resonance imaging (MRI). It was confirmed again with intraoperative findings of compression of cisternal portion of the facial nerve by the meatal loop of the anterior inferior cerebellar artery (AICA) and absence of any offending vessel in the REZ of the facial nerve. Immediate disappearance of lateral spread response (LSR) after decompression and resolution of spasm after the operation again validated that HFS in the current patients originated from the vascular compression of distal, cisternal portion of the facial nerves. According to our literature review of 64 patients with HFS caused by distal neurovascular compression, distal compression can be classified by pure distal neurovascular compression (31 cases, 48.4%) and double compression (both distal segment and the REZ of the facial nerves, 33 cases [51.6%]) according to the presence or absence of simultaneous offender in the REZ. Eighty-four percent of 64 identified distal offenders were the AICA, especially its meatal and postmeatal segments. Before awareness of distal neurovascular compression causing HFS and sophisticated MRI imaging (before 2000), the rate of reoperation was high (58%). Preoperative MRI and intraoperative monitoring of LSR seems to be an essential element in determination of real offending vessel in MVD caused by distal offender.

## 1. Introduction

Primary HFS is generally regarded as the result of hyperexcitability of the facial nerve and its nucleus caused by vascular compression of the facial nerve at its root exit zone (REZ) [1–13]. However, HFS caused by vascular compression in the distal portions of the facial nerve has been sporadically reported [14–23]. In the reports of HFS caused by vascular compression of the distal, cisternal portion of the facial nerve, distal neurovascular compression has mostly been identified by intraoperative finding during repeated MVD for recurrences or failures after MVD [9, 15–17]. However, preoperative identification of distal neurovascular conflict is now increasing since introduction of sophisticated MRI examination in HFS [18, 19, 24–26].

We report two patients with typical HFS caused by pure cisternal neurovascular compression of the facial nerve by the meatal and postmeatal segments of the AICAs. We summarized a detailed review of literature regarding HFS caused by distal neurovascular compression according to the pattern of the offending vessels. According to literature review of HFS caused by distal neurovascular compression, distal neurovascular compression can be classified by pure neurovascular compression in distal, cisternal portion by a single artery (mostly by the anterior inferior cerebellar artery [AICA]) and double compression (both REZ and the distal portion) by a single arterial loop or two different offending arteries (e.g., AICA and the posterior inferior cerebellar artery [PICA], or AICA and the vertebral artery [VA]).

In determination of true distal offender, intraoperative monitoring of lateral spread response (LSR) was helpful. LSR is an abnormal muscle response demonstrated by EMG recordings from mimic muscles that are innervated by a different branch of the facial nerve [3, 27–29]. When the offending vessel is moved off the REZ of the facial nerve, LSR disappears instantly in most patients [3, 22, 27–29]. Although seeking elimination of LSR in all patients after verification of complete decompression is not recommended [30], assuring disappearance of LSR could enhance surgical success rate without missing possible hidden offenders around the REZ. Although importance of LSR monitoring during primary and repeated MVDs has been reported [11, 12, 27–29, 31–33], its role in determination of distal offender has been rarely addressed [19].

## 2. Case Report

### 2.1. Case 1

**Figure 1:**
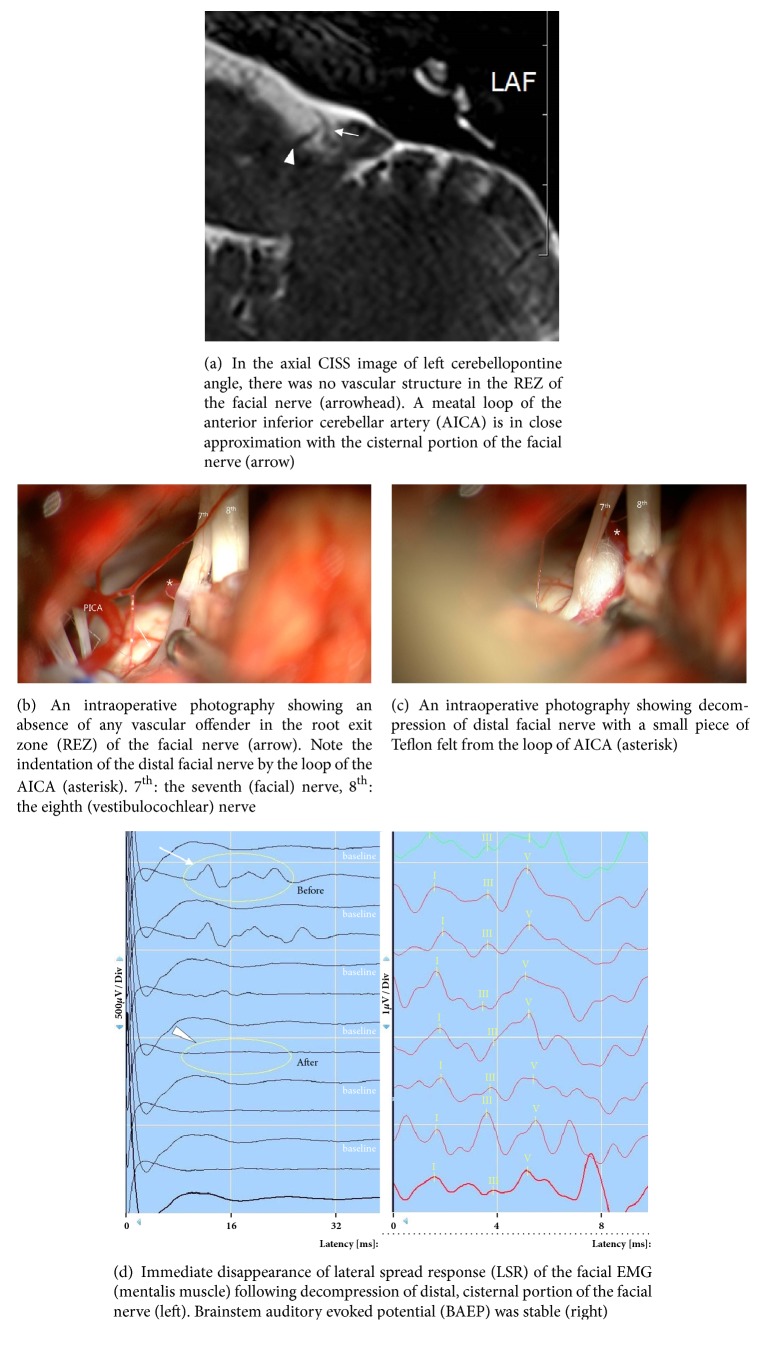
Magnetic resonance imaging (MRI) and intraoperative findings of hemifacial spasm (HFS) caused by distal offender (the meatal loop of AICA, case 1).

A 50-year-old male patient presented with a 2-year history of left-sided typical HFS. Painless irregular clonic contraction of the facial muscles began initially in the orbicularis oculi muscle of the lower lid. It gradually spread to other muscles innervated by the facial nerve on the left side of the face, including platysma. The paroxysm was induced or aggravated by emotional tension, stress, and voluntary and reflexive movements of the face. He had significant difficulty in his work and social life despite 2 times of botulinum toxin injection. Medical treatment with carbamazepine (up to 600 mg) and baclofen (30 mg) was not effective. He was referred for surgical treatment. His medical history was unremarkable. His physical and neurologic examinations were normal, including hearing. No tinnitus or discernible noise heard in his left ear was found. Only typical nature of clonic hemifacial spasm was evident. Abnormal synkinesis between the orbicularis oculi and orbicularis oris muscles was found by the electromyographic examination of the blink reflex. Despite typical HFS, there was no discernible vascular structure in the REZ of left facial nerve (Figure 1(a)). However, a meatal loop of AICA abutting to the cisternal portion of the facial nerve was found.

Under the impression of HFS caused by neurovascular compression of distal facial nerve, standard microsurgical procedure was performed as described previously [5, 7, 10]. In addition to intraoperative monitoring of BAEPs, LSR, which is an abnormal muscle response demonstrated by EMG recordings from mimic muscles that are innervated by a different branch of the facial nerve [3], was also monitored throughout the operation. The entire course of the facial nerve and offending arteries were exposed under microscopic vision. Upon exposure of the REZ of the facial nerve, there was no offending vessel in the REZ as expected (Figure 1(b)). The distal, cisternal segment of the facial nerve was found to be bent by a meatal loop of the AICA (Figure 1(b)). A small piece of Teflon felt was interposed between the facial nerve and the meatal loop of the AICA with extreme care not to stretch the internal auditory artery and the distal facial nerve (Figure 1(c)). After interposition of Teflon felt, LSR immediately disappeared and BEAP was stable also (Figure 1(d)). The closure of the dura and wound was performed in routine manner. The HFS resolved completely following the surgery. The postoperative course was uneventful with no signs of facial weakness or hearing impairment by pure-tone audiometry. No recurrence of HFS or neurologic sequelae was evident at a 12-month follow-up.

### 2.2. Case 2

**Figure 2:**
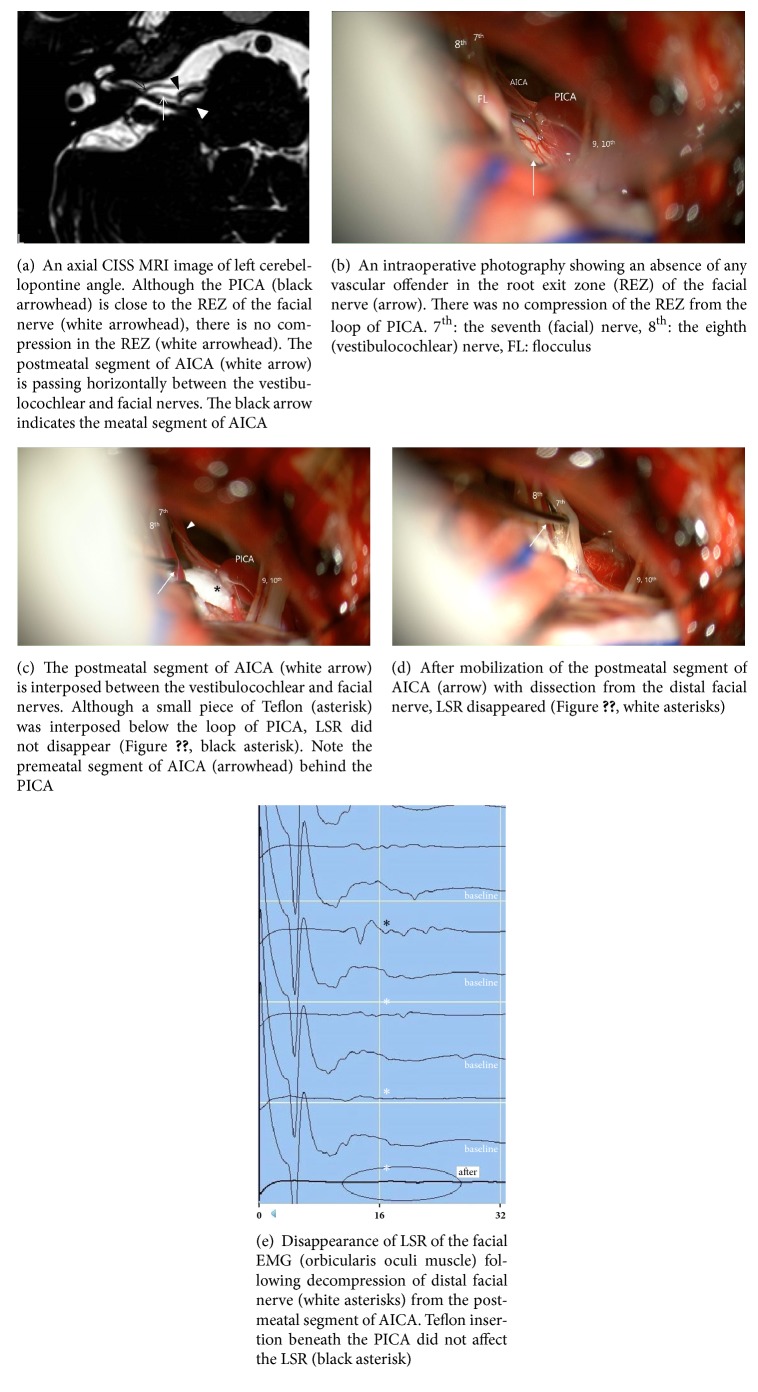
Magnetic resonance imaging (MRI) and intraoperative findings of the right-sided hemifacial spasm (HFS) caused by distal offender (the postmeatal segment of AICA, case 2).

A 58-year-old female patient presented with a 1-year history of right-sided typical HFS. The nature of spasm was similar to case 1 and identified as typical HFS. It progressively worsened and did not respond to medical treatment and botulinum toxin was effective only for three months. She wanted to have a definitive treatment and transferred to our department. Her neurologic examination was normal except painless irregular clonic contraction of the facial muscles, consistent with typical HFS. In the MRI, although the PICA passed around the REZ of the facial nerve, it did not compress the REZ (Figure 2(a)). The postmeatal segment of AICA coursed between the vestibulocochlear and facial nerves. Under suspicion of HFS by distal neurovascular compression, MVD was performed with intraoperative monitoring of LSR and BAEP. As expected, the PICA had no association with the REZ or attached segment of the facial nerve (Figure 2(b)). The postmeatal segment of AICA was interposed between the vestibulocochlear and facial nerves and adhered to the distal cisternal segment of the facial nerve. It was carefully separated from the facial nerve and 2 thin leaflets of Teflon were interposed between the postmeatal AICA and the facial nerve (Figure 2(c)). Disappearance of LSR was confirmed within 2 minutes (Figure 2(d)). After awakening from anesthesia, the spasm disappeared. Postoperative course was uneventful with any facial weakness or hearing impairment by pure-tone audiometry. She discharged at the fifth postoperative day and no recurrence was found at 6 months postoperatively.

## 3. Discussion

### 3.1. Primary Hemifacial Spasm Caused by Distal Offender and Its Preoperative Identification

According to our literature review (Table 1), the problems associated with MVD for primary HFS caused by distal neurovascular compression are preoperative identification of distal compression and high rate of reoperation. In the period (1990s and early 2000s) when a detailed MRI examination and intraoperative monitoring of LSR were not popular, distal neurovascular compression was confirmed only during the repeated operation for spasm recurrence or surgical failure [15], or an intraoperative finding of only distal offender with its absence in the REZ [14, 16, 17]. Indeed, 7 (58%) of the 12 reported cases of distal neurovascular compression before 2000 needed a reexploration to figure out the presence of distal compression [14–17]. The description of the outcome of the first MVDs on HFS with distal offenders was often vague, without distinguishing persistence or recurrence of HFS. Recurrence represents symptoms reappearing postoperatively after a symptom-free interval of more than 1 year [34]. When there is no improvement or worsening of symptoms after one year postoperatively, it is called “incomplete cure” or persistence [34, 35]. Reasons for an unsuccessful MVD include incomplete decompression by not identifying the true culprit vessels, presence of a previously unidentified secondary offending vessel, or implant compression/migration against the facial nerve [36–38].

In addition, LSR monitoring was not performed in all 12 cases of distal neurovascular compression reported before 2000 [14–17]. Lack of knowledge about the utility of LSR monitoring during MVD in that era might have resulted in a high rate of failure and recurrence (58%) in HFS caused by distal offenders. Although true value of LSR monitoring is still in doubt, a meta-analysis study found that the chance of a cure if LSR was abolished during surgery was 4.2 times greater than that if LSR persisted [11]. Because the reports specifically addressing HFS associated with distal offenders are rare, the results of LSR monitoring in this situation are largely unknown.

Sophisticated MRI techniques for lower cranial nerves in the cerebellopontine angle and the internal auditory canal were introduced in late 1990s and were popularized since early 2000s [24, 39]. The three-dimensional constructive interference of steady-state sequence (3D CISS) and three-dimensional fast imaging employing steady-state acquisition (3D FIESTA) technique [24, 26, 39, 40] provided superior visualization of neurovascular relationships with the excellent CSF-nerve contrast and high spatial resolution. They have enabled preoperative identification of HFS caused by distal neurovascular compression [18, 19, 24–26]. However, even with well-visualization of the neurovascular structures in and around the REZ and distal facial nerve, there are many asymptomatic neurovascular contacts in the facial nerve [19, 24–27, 41]. Multiple offenders are also common [19, 23, 27]. Multiple compression by a single artery, such as compression of the REZ by premeatal segment of the AICA and distal compression by its distal meatal segment, has also been reported [23]. Therefore, unless there is no definite arterial offender in the REZ in patients with typical HFS associated with only single, suspicious offending vessel in the distal facial nerve, it is difficult to make a preoperative diagnose of HFS caused by distal neurovascular conflict. Furthermore, HFS associated with venous offender was also reported [42–44].

In our review of distal neurovascular compression causing HFS, it was found to occur in 2 types according to the presence or absence of additional vascular offenders in the REZ. Therefore, it can be assorted into the pattern of distal compression: (1) pure distal compression by single offender without any offender in the REZ (PDC type), (2) double compression in both distal cisternal portion and the REZ of the facial nerve (DC type). A total of 64 cases of surgically proven patients with HFS caused by distal compression were identified, including current 2 cases (Table 1). Thirty-one cases (48.4%) were found to be PDC type and 33 cases (51.6%) were DC type. The incidence of distal neurovascular compression varied significantly according to the reports, ranging from 5.9% to 1 out of 753 patients [9, 22, 23].

With development of new techniques of MRI examinations and spread of knowledge regarding distal neurovascular compression in HFS, rate of reoperation in distal compression decreased significantly since 2000. Among 52 reported cases of distal compression after 2000, 1 of 25 PDCs (4%) and 7 (25.9%) of 27 DCs needed repeated operations. However, the rate of reoperation in DC type is high and still poses significant problem during MVD for HFS [12, 22, 23]. Difficulty in determination of real culprit offender in the presence of double compression in both distal segment and the REZ of the facial nerve (DC type) during the microsurgical operation has been repeatedly stressed by several authors [12, 15, 22, 23]. Furthermore, importance of awareness of DC type was suggested as early as 1991 by Nagahiro et al. [15] as “sandwich type”. Zheng et al. [23] stressed it as “cross-type” compression again. Although the uncertainty associated with HFS caused by PDC type decreased significantly with preoperative identification and intraoperative LSR monitoring as shown in the current 2 cases, it seems that those with DC type still pose significant surgical challenges during MVD.

### 3.2. Meatal and Postmeatal Segments of the Anterior Inferior Cerebellar Artery (AICA) as Distal Offender

Fifty-four (84.3%) identified distal offenders among 64 cases of HFS caused by distal compression were the AICA and seven (10.9%) were the PICA. Since the early reports by Yeh et al. [14] and Nagahiro et al. [15], the AICA was the main distal offender causing HFS. Among the four segments (anterior pontine, lateral pontine, flocculonodular, and cortical) of the AICA [45], the second (lateral pontine) segment gives rise to the nerve-related branches that course near or within the internal acoustic meatus in close relationship to the facial and vestibulocochlear nerves. This segment is divided into premeatal, meatal, and postmeatal parts, depending on their relationship to the porus acusticus. The premeatal segment of the AICA courses around the brainstem to reach the facial and vestibulocochlear nerves and the anterior edge of the meatus. Most (46 of the 56, 82%) of the premeatal segments were anteroinferior to the nerves and, therefore, they are common offenders in the REZ of the facial nerve [45]. The meatal segment, located in the vicinity of the internal acoustic meatus, often forms a laterally convex loop directed toward the meatus. The majority of the meatal loops coursed in a horizontal plane above or below the nerves, but some, mostly those passing between the facial and vestibulocochlear nerves, course in a vertical or oblique plane [45]. These meatal and postmeatal segments of the AICA passing between the facial and vestibulocochlear nerves, as shown in current report, have been repeatedly identified and described as “interposed AICA between the facial and vestibulocochlear nerves” [12, 14–19, 22].

### 3.3. Role of Lateral Spread Response during MVD for HFS Caused by Distal Offender

When there is a single offending artery in the distal segment of the facial nerve (PDC type) and LSR is consistent after exposure of the REZ of the facial nerve during MVD, it can be a reliable means of confirming the culprit vessel. Even if there are dual offending vessels along the facial nerve, both in the REZ and distal segment (DC type), LSR may be a reliable indicator of adequate decompression [31–33]. If LSR disappears immediately after initial decompression of the REZ, further decompression of distal facial nerve may not be needed. However, if LSR persists despite adequate decompression, deciding to perform distal decompression is difficult and is at the surgeon's discretion.

## 4. Conclusions

Two cases of typical HFS caused by distal neurovascular compression of the meatal and postmeatal segments of AICA are presented. According to literature review, HFS caused by distal neurovascular compression could be classified into a pure distal compression (PDC type) and those with double compression in both the REZ and distal segment of the facial nerves (DC type). The meatal and postmeatal segments of AICA are the most common distal offenders. Preoperative suspicion of distal compression with MRI examination and intraoperative verification of distal offender in absence of offenders in the REZ of the facial nerve, combined with intraoperative monitoring of LSR, seems to be essential element in MVD for HFS caused by distal offenders.

## Figures and Tables

**Table 1:** Summary of reported cases of hemifacial spasm caused by distal compression of the facial nerve.

Authors,	Number	Age/sex	Side	Confirmation	Findings, offender			LSR	Relief	Cx.	Classification
Year	cases		spasm	distal comp.	OR	at REZ	at cisternal	usefulness	spasm		(PDC/DC)
Yeh, et al. [14]	2	52/f, 57/m	lt	intraoperative	once	x	AICA interposed	n/a	excellent	none	2 cases, PDC
1981											interposed AICA between 7^th^, 8^th^

Nagahiro et al. [15]	2	n/s	rt	case 1; intraop.	1^st^ OR;	PICA	-	n/s	recur	none	1 case, DC
1991					2^nd^ OR;	-	AICA, meatal	n/a	immediate	none	“Sandwich type”, stressed
				case 2; intraop.	1^st^ OR;	AICA, premeatal	-	n/a	recur	none	1 case, DC
					2^nd^ OR;	-	AICA, postmeatal	n/a	immediate	none	interposed AICA between 7^th^, 8^th^

Fukuda, et al. [16]	1	61/f	lt	intraoperative	1^st^ OR;	PICA	-	failure	none		1 case, PDC
1997					2^nd^ OR;	-	AICA, meatal	useful (+/ -)	immediate	none	interposed AICA between 7^th^, 8^th^

Ryu, et al. [17]	7	36-73	rt(3)/lt(4)	case 1; intraop.	once	x	PICA	n/a	resolved		4 cases, PDC, (1 reop.)
1998		f:m=6:1		case 3; intraop.	Once	x	AICA	n/a	resolved		
				case 5; intraop.	1^st^ OR;	AICA	-	n/a	failure		
					2^nd^ OR;	-	AICA	n/a	resolved	hearing loss	
				case 7; intraop.	Once	x	AICA	n/a	resolved		
				case2; intraop.	1^st^ OR;	AICA	AICA	n/a	failure		3 cases, DC (all reop.)
					2^nd^ OR;	-	AICA	n/a	resolved	7^th^	
				case 4; intraop.	1^st^ OR;	AICA	-	n/a	recur, 3 mo	7^th^	
					2^nd^ OR;	x	AICA	n/a	resolved		
				case 6; intraop.	1^st^ OR;	PICA	-	n/a	recur, 3 yr		
					2^nd^ OR;	-	PICA	n/a	resolved		

Onoda, et al. [18]	2	51/f (case 1)	rt	preop. MRI	case 1;	x	AICA	n/a	resolved	none	2 cases, PDC
2006		71/f (case 2)	rt	preop. MRI	case 2,	x	AICA	n/a	resolved	none	interposed AICA between 7^th^, 8^th^

Campos-Benitez, et al. [9]	4	n/a	n/a	intraop.	n/s	x	AICA (3)/ PICA (1)	n/a	n/a	n/a	4 cases, PDC
2008											3% incidence of distal comp.

Kawashima et al.[19]	1	50/f	lt	preop. MRI	once	x	AICA, meatal	useful (+/-)	immediate	none	1 case, PDC
2009											interposed AICA between 7^th^, 8^th^

Chang, et al. [20]	14	35-66	n/a	preop. MRI	n/a	x	AICA (8)/ PICA(4)	n/s	excellent	7^th^ palsy (1)	12 cases, PDC
2010		f:m=10:4					/V(1)/ multi (1)		71.4%		incidence 14 of 2137

Zhong, et al. [12]	7	n/a	n/a	intraop.	1^st^ OR;	AICA	-	n/s	no relief	-	7 cases, DC, “cross-type” comp.
2010					2^nd^ OR;	-	AICAs, zone 4 (7)	n/s	excellent	7^th^ palsy (1)	by all missed AICAs (reop.)

Li, et al. [22]	1	50/m	n/s	intraop.	1^st^ OR;	AICA	-	n/s	no relief	-	1 case, PDC
2010					2^nd^ OR;	-	AICA, meatal	useful (+/-)	immediate	7^th^ palsy	incidence of distal comp.; 1/753

Zheng, et al. [23]	21	48.4	rt(10)/lt(11)	intraop.	once	AICA (20)	AICA (20)	useful (+/-)	resolved	tinnitus (3)	20 DCs/1 PDCs, “Cross-type”
2011		f(15)/m(6)			once	x	AICA (1)	useful (+/-)	resolved	hearing (3)	incidence, 21 of 355 (5.9%)

Current case	2	50/m	lt	preop. MRI	once	x	AICA, premeatal	useful (+/-)	immediate	none	2 cases, PDC
2018		53/f	rt	preop. MRI	once	x	AICA, postmeatal	useful (+/-)	immediate	none	interposed AICA between 7^th^, 8^th^

AICA: anterior inferior cerebellar artery, comp.: compression, Cx.: complications, HFS: hemifacial spasm, m: male, f: female, intraop.: intraoperatively, rt: right, LSR: lateral spread response, lt: left, MRI: magnetic resonance imaging, n/a: not available, n/s: not specified, OR: operation, PICA: posterior inferior cerebellar artery, preop.: preoperative, reop.: reoperation, 7^th^: the seventh (facial) nerve, V: vein, (+/-): negative conversion of lateral spread response after distal decompression. Classification of distal compression: PDC: single-artery, pure distal compression, DC: double compression.
